# When the Skin Tells Another Story: Incidental Epidermolysis Bullosa in an Adult Hospitalized for Chronic Respiratory Symptoms

**DOI:** 10.7759/cureus.110269

**Published:** 2026-06-04

**Authors:** Enrique Acosta García, Jacqueline Castillo Martínez, Krysta P Carranza Ambriz, Edwin Maya Talamantes, Mario Lozano Aguilar

**Affiliations:** 1 Internal Medicine, Hospital General ISSSTE Fray Junípero Serra, Tijuana, MEX; 2 Dermatology, Hospital General ISSSTE Fray Junípero Serra, Tijuana, MEX; 3 Research, Hospital Universitario Dr. José Eleuterio González, Monterrey, MEX

**Keywords:** chronic cough, dermatologic examination, epidermolysis bullosa, incidental diagnosis, skin fragility, subepidermal blister

## Abstract

Epidermolysis bullosa (EB) comprises a heterogeneous group of inherited blistering disorders characterized by skin fragility and trauma-induced blister formation. Although EB is typically recognized in childhood, milder phenotypes may remain underdiagnosed in adults and may be identified incidentally during evaluation for unrelated medical conditions. A 47-year-old man was admitted for chronic respiratory symptoms and a history of prior granulomatous infections. During a routine physical examination, extensive chronic blistering skin lesions were identified incidentally. Inpatient evaluation ruled out reactivation of pulmonary tuberculosis, pneumonia, and coccidioidomycosis, with chest computed tomography demonstrating only residual apical fibrosis. Dermatologic evaluation raised suspicion for EB. Subsequent outpatient skin biopsy revealed a subepidermal blister with minimal inflammatory infiltrate compatible with EB. This case highlights the diagnostic value of comprehensive physical examination and emphasizes the importance of multidisciplinary evaluation when rare dermatologic conditions are encountered in adult inpatients.

## Introduction

Epidermolysis bullosa (EB) comprises a heterogeneous group of rare inherited dermatoses characterized by structural defects at the dermoepidermal junction, resulting in marked skin fragility and blistering following minimal mechanical trauma [1,2]. As the prototypical disorder of skin fragility, EB exhibits a broad phenotypic spectrum ranging from localized forms to severe generalized disease with extracutaneous involvement, often associated with significant morbidity, including infection, scarring, and increased mortality in advanced cases [2].

EB is classically classified into four major types--EB simplex, junctional EB, dystrophic EB, and Kindler syndrome--based on the level of tissue cleavage and underlying molecular features [3,1]. In addition, multiple subtypes have been described, including EB pruriginosa, associated with mutations in the COL7A1 gene that affect collagen VII and anchoring fibrils [4]. Other disorders characterized by skin fragility should also be considered in the differential diagnosis due to overlapping clinical features [2].

The diagnosis of EB is initially based on clinical suspicion and requires confirmation through specialized techniques such as immunofluorescence mapping, electron microscopy, and genetic testing, which allow accurate subtype classification and guide clinical management [5]. However, access to these diagnostic tools remains limited in many settings, leading to reliance on clinical evaluation and potential underclassification of cases [6].

Despite its rarity, with reported prevalence estimates of approximately 6.77 cases per million population, EB represents a condition with significant clinical and socioeconomic burden [6]. Current therapeutic approaches are mainly supportive, as definitive treatments such as gene and cell-based therapies remain under development and face important implementation challenges [7]. Furthermore, EB has a substantial impact on patients’ quality of life and mental health, with a high prevalence of depressive symptoms, highlighting the need for comprehensive and multidisciplinary care strategies [8].

## Case presentation

A 47-year-old man was admitted for evaluation of a one-year history of predominantly morning cough with intermittent improvement and recurrence. The cough initially produced clear sputum but later progressed to purulent expectoration. Symptoms were associated with diaphoresis, dyspnea on moderate exertion, fatigue, asthenia, and generalized weakness. He denied fever, chills, headache, or involuntary weight loss.

His past medical history was notable for miliary tuberculosis two years earlier, with limited documentation available, and pulmonary coccidioidomycosis that had been diagnosed one year earlier, which was treated with fluconazole 100 mg daily for four months with reported clinical improvement. Surgical history included an open appendectomy approximately 30 years prior. The patient reported a penicillin allergy and denied tobacco use, alcohol consumption, illicit drug use, prior blood transfusions, fractures, or exposure to biomass smoke.

On admission, vital signs were stable: blood pressure (136/84 mmHg), heart rate (97 bpm), respiratory rate (18/min), temperature (36.2°C), and oxygen saturation (95%) on room air. Weight was 67 kg and height was 163 cm (BMI 25.3 kg/m²). Physical examination revealed an ectomorphic habitus, generalized pallor, and decreased skin hydration. Neurological examination was normal with a FULL Outline Of UnResponsiveness (FOUR) score of 16/16.

Pulmonary auscultation revealed diminished vesicular breath sounds bilaterally without crackles or wheezes. Cardiac examination demonstrated a regular rhythm without murmurs. The abdomen was soft and non-tender, and the extremities were well perfused.

**Figure 1:**
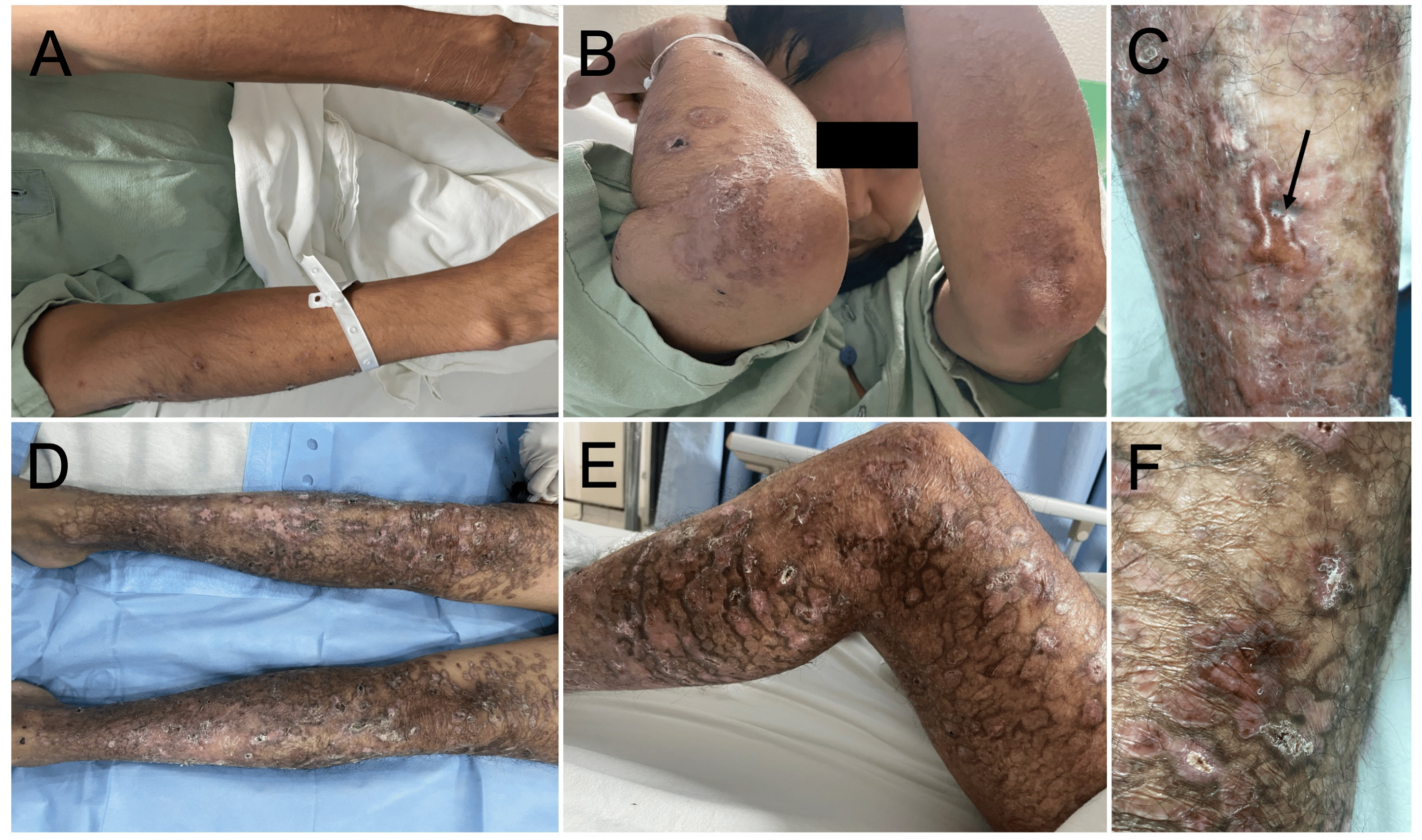
Cutaneous manifestations compatible with epidermolysis bullosa. (A) Upper extremities showing scattered hyperpigmented macules and atrophic scars. (B) Elbow involvement with xerosis, focal lichenification, and healed lesions with residual pigmentation. (C) Tense blister over the left tibial tuberosity (black arrow). (D) Bilateral lower-extremity involvement demonstrating extensive polymorphic lesions with post-inflammatory hyperpigmentation, atrophic scarring, and superficial erosions in different stages of healing. (E) View of the leg showing multiple hyperpigmented plaques, atrophic scars, and punctate hemorrhagic crusts with mild perilesional erythema. (F) Close-up view of the lower extremity showing ulcerated nodules and crusted plaques on a background of post-inflammatory hyperpigmentation and scarring.

Dermatologic examination revealed bilateral polymorphic lesions on the lower extremities characterized by post-inflammatory hyperpigmentation, atrophic scarring, superficial erosions in different stages of healing, diffuse xerosis with focal lichenification over pressure areas, and punctate hemorrhagic crusts with mild perilesional erythema. A tense blister was observed over the left tibial tuberosity. These findings were clinically compatible with EB (Figure 1).

**Figure 2:**
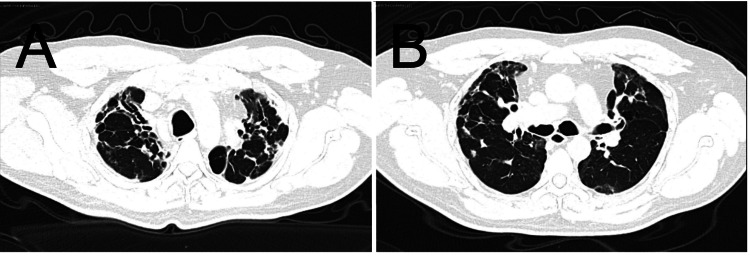
Chest CT findings. (A) Axial chest CT demonstrating residual fibrotic changes in the upper lobes consistent with prior granulomatous infection. (B) Additional axial section showing residual apical fibrosis without cavitation or consolidation, with no radiologic evidence of active pulmonary infection.

Diagnostic evaluation focused initially on excluding reactivation of granulomatous infections. Three serial sputum smears for acid-fast bacilli were negative. Serologic testing for coccidioidomycosis (IgG and IgM) was negative. Non-contrast chest CT demonstrated residual apical fibrotic changes without cavitation or consolidation (Figure 2). During a five-day hospital stay, the patient remained afebrile and clinically stable, without leukocytosis or leukopenia and with negative procalcitonin levels.

The dermatology consultation recommended skin biopsy for diagnostic confirmation. A biopsy was subsequently performed on an outpatient basis from a lesion on the anterior aspect of the leg.

**Figure 3:**
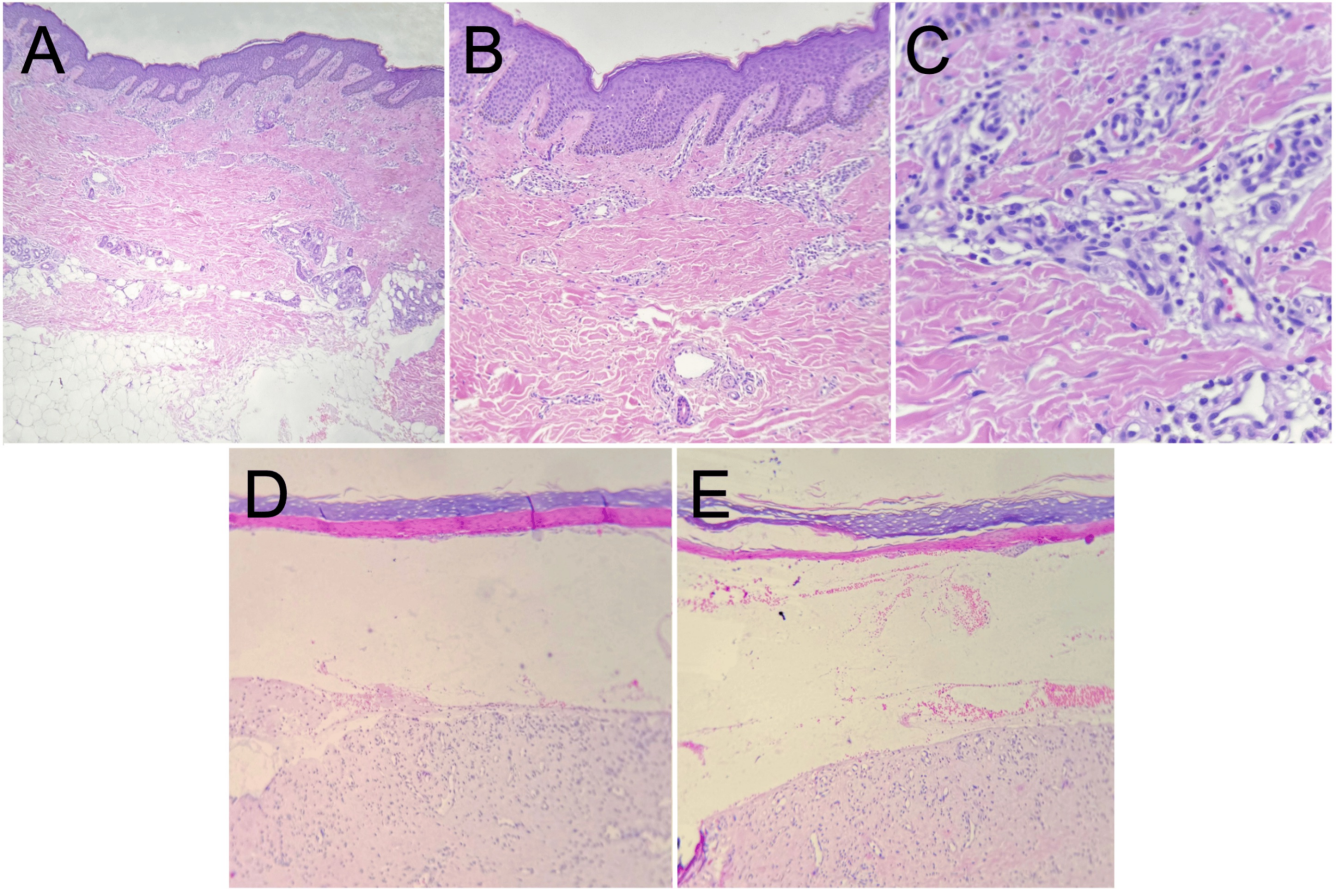
Histopathological findings compatible with epidermolysis bullosa (hematoxylin and eosin stain). (A) Low-power view showing epidermal flattening with loss of normal rete ridge architecture. (B) Laminar hyperkeratosis with flattening of the rete ridges. (C) Superficial dermis with minimal perivascular lymphohistiocytic inflammatory infiltrate. (D) Subepidermal blister formation with separation between epidermis and dermis. (E) Blister cavity containing serous material and erythrocytes.

Histopathological examination revealed laminar hyperkeratosis of the stratum corneum and flattening of the rete ridges. A subepidermal blister containing serous material and erythrocytes was identified without significant inflammatory infiltrate in the blister cavity. The superficial dermis showed minimal perivascular lymphohistiocytic inflammatory infiltrate. No evidence of malignancy was observed. These histopathological findings supported the clinical suspicion of EB (Figure 3).

## Discussion

EB represents a heterogeneous group of inherited blistering disorders caused by pathogenic variants affecting structural proteins of the dermoepidermal junction. These alterations lead to mechanical fragility of the skin and mucous membranes, resulting in blister formation following minimal trauma. Although EB is commonly recognized during infancy or childhood, milder phenotypes may persist into adulthood or remain undiagnosed in individuals with limited access to specialized dermatologic care [1,2,6].

This case illustrates how EB may be identified incidentally during routine physical examination in an adult hospitalized for an unrelated medical condition. Similar delayed or incidental diagnoses have been described in patients with less severe phenotypes, in whom clinical manifestations may be subtle or misattributed to other chronic dermatoses [2,6]. The patient’s dermatological findings--including chronic polymorphic lesions with post-inflammatory hyperpigmentation, atrophic scarring, and erosions in different stages of healing--are consistent with the clinical heterogeneity and chronic evolution reported in EB cohorts [1,4].

Histopathological evaluation further supported the diagnosis, demonstrating a subepidermal blister with minimal inflammatory infiltrate. These findings are in line with previously described histologic patterns of inherited blistering disorders and help distinguish EB from autoimmune bullous diseases, which typically show more prominent inflammatory infiltrates and immunologic features [6]. This distinction is well established and underscores the structural rather than inflammatory nature of EB [1,2].

In adults presenting with chronic blistering and erosive lesions, the differential diagnosis includes bullous pemphigoid, porphyria cutanea tarda, dermatitis artefacta, and chronic prurigo with excoriations. Previous reports emphasize that autoimmune bullous diseases may present with tense bullae but generally lack the longstanding scarring and pigmentary changes characteristic of EB [6]. Likewise, variants such as EB pruriginosa may clinically resemble chronic pruritic dermatoses, further complicating diagnosis and delaying recognition [4,7].

Recognition of EB in hospitalized patients has important practical implications. Individuals with EB are at increased risk of iatrogenic skin injury from adhesive devices, blood pressure cuffs, and repeated venipuncture. Preventive measures include the use of non-adhesive dressings, gentle handling of the skin, protective padding over pressure points, and early dermatology involvement. Furthermore, disruption of the skin barrier predisposes patients to secondary infections, and clinicians should maintain a low threshold for evaluating superinfection when local or systemic signs of infection are present [8].

Management of EB in adults requires a multidisciplinary approach. In addition to minimizing skin trauma and using non-adhesive dressings, comprehensive wound care, infection surveillance, and adequate nutritional support are important components of management. Long-term follow-up should also include monitoring for extracutaneous complications and cutaneous squamous cell carcinoma, particularly in patients with dystrophic forms of the disease.

## Conclusions

EB may be identified incidentally in adult patients hospitalized for unrelated medical conditions. Recognition of characteristic dermatologic findings during routine physical examination is essential to prevent misdiagnosis and minimize the risk of iatrogenic skin injury. Early multidisciplinary evaluation, including dermatology consultation and appropriate follow-up, plays a key role in ensuring accurate diagnosis and optimizing long-term management of patients with suspected EB.
